# Alcohol Intoxication in the Academic Emergency Department: Epidemiology and Facility-Fee Financial Impact

**DOI:** 10.5811/westjem.43575

**Published:** 2025-10-03

**Authors:** Eric Legome, William Bonadio, Michael Redlener, Elyse Lavine, Avah Mealy, Samuel E. Sondheim

**Affiliations:** * Icahn School of Medicine at Mount Sinai, Department of Emergency Medicine, New York City, New York; † Icahn School of Medicine at Mount Sinai, Center for Healthcare Readiness, New York City, New York

## Abstract

**Introduction:**

Alcohol intoxication is a common patient presentation to urban emergency departments (ED). There is limited data on the healthcare financial impact of caring for alcohol-intoxicated patients in the ED. In this study we examined the facility-based financial billings and collections related to ED visits for alcohol intoxication.

**Methods:**

Using a retrospective cohort analysis of two large, urban EDs, with a combined yearly census of approximately 150,000 patient visits, we included all encounters between June 2018–December 2021 with a discharge diagnosis consistent with acute alcohol intoxication. We reviewed records of patient encounters with a final diagnosis consistent with acute alcohol intoxication who only had minimal or no interventions performed, implying the visit was solely consistent with acute alcohol intoxication. We reviewed the facility charges of these patients, along with insurance status and average payment by status to understand the financial impact.

**Results:**

Of 495,436 patient presentations to the EDs during the study period, 13,454 met study criteria (2.7% of total patients). Patient length of stay in the ED had an average of 254 minutes and median of 240 minutes. In total, this cohort of patients occupied ED beds for 56,505 hours cumulatively, or an average of 43.2 bed hours per day for alcohol intoxication-related visits, representing 3.14% of all ED bed hours across both sites. The majority of patient encounters were billed as a level 3 facility code (76%). Facility charges for the cohort totaled $22,590,000. The estimated reimbursement based on the percentage reimbursed by payor mix was $1.7 million (7.5%), or an average of $126 per patient visit—less than one quarter of the general average visit collection.

**Conclusion:**

Patients with acute alcohol intoxication and no other complaints are a minority of ED patients, yet their care results in substantial charges and ED resources. Based on the known facility collection rates per insurer, the weighted prevalence of insurers among this cohort yields an estimated collection rate of 7.5%. Opportunities to provide proven alcohol-related interventions should consider the unreimbursed costs of these visits when determining cost effectiveness.

## INTRODUCTION

Acute alcohol intoxication is a common patient presentation to urban emergency departments (ED). Given space and staffing constraints to safely monitor these patients, particularly those at high fall risk, these encounters can be expensive and resource intensive.^1^ Additional related challenges in the ED include increased crowding, longer length of stay (LOS), and abusive behavior toward staff and other patients.^2^–^6^ Alcohol use disorder in the US has a significant financial impact on the healthcare system at large, with estimated costs of $24.6 billion in 2006, of which only 10.3% were paid for by alcohol users and their families.^7^ Similar estimates from 2010 placed healthcare costs at $28.4 billion.^8^ From 2006 to 2014, the rate of acute alcohol-related ED visits increased 40% from 720.9 to 1,009.6 per 100,000 population.^9^

Undoubtedly, a large portion of the overall healthcare costs for these visits is attributable to emergency care, as it is the main safety net for receiving patients while acutely intoxicated or with complications related to alcohol consumption. Yet there is little ED-specific literature describing the facility-based financial charges, insurance status, and financial implications of alcohol-intoxicated patients in the ED. In this paper we provide specific financial estimates about patients who present to the ED with acute alcohol intoxication but do not require evaluation otherwise.

## METHODS

In this retrospective cohort analysis of two large, academic, urban EDs within the same health system, with a combined yearly census of approximately 150,000 patient visits, we included all patient encounters between June 2018–December 2021 with a discharge diagnosis consistent with acute alcohol intoxication via a query through the electronic health record. We used *International Classification of Diseases, 10^th^ Rev.* (ICD 10) F10.1–9 codes encompassing alcohol use and dependence (Table 1). As there is not a definitive code that every physician uses at discharge, we included those generally used for the acutely intoxicated patient. We excluded patients with ICD-10 codes that included other potential concomitant issues that may have required separate treatment, such as withdrawal, delirium, or another dysfunction. Patients were also excluded if they were admitted, had any additional lab testing on the same visit (excluding COVID-19 or blood glucose testing) or imaging studies, or had any treatment beyond an immunization administration (TDAP or influenza) strongly suggesting they had presentations or complaints for etiologies other than purely alcohol-related or that it was arrived at as a diagnosis after testing or interventions. We excluded mixed intoxications, although as diagnoses were generally made by patient or history/presentation to emergency medical services, this likely underestimated co-ingestions. In most cases, the diagnosis of acute alcohol intoxication was a clinical diagnosis.

The current practice at these facilities does not include routine testing for alcohol level or toxicological screening without clinical indications. This is a common practice in emergency medicine. In general, if the history is consistent with findings (eg, patient admits to alcohol consumption, odor of alcohol, and clinical picture, etc), and the patient clinically improves over time, alcohol testing is not required. If the patient fails to improve, or if there is evidence of head trauma or concern for altered mental status that is not sufficiently explained by alcohol, further testing is often required.^10^ Due to the retrospective nature of this study and the change in professional billing groups during the study time frame, we were unable to obtain data on professional charges. Therefore, the financial charges are related solely to ED facility fees. As a proxy to quantify the clinical impact, we reviewed LOS for this cohort of patients as well. Charts were abstracted per prior standards, including case selection criteria and variable definitions.^11^ This research was approved by the institutional review board.

**Population Health Research Capsule**

What do we already know about this issue?

*Alcohol intoxication-related visits pose significant challenges to EDs. They require significant resources to ensure patient and staff safety and often face significant costs.*

What was the research question?

*How can we quantify the costs and reimbursements associated with alcohol-related ED visits?*

What was the major finding of the study?

*Alcohol-related ED visits comprised over 3% of all ED bed hours, with the estimated reimbursement less than one quarter of the general average visit collection.*

How does this improve population health?

*The heavy resource use of alcohol-related visits to the ED often yields minimal financial collections.*

## RESULTS

Of 495,436 patient presentations to the two EDs during the study period, 16,526 (3%) visits met criteria of a diagnosis of acute alcohol intoxication. Of these, 10,800 were unique medical record numbers with ED visits per patient ranging from 1–130 visits over that period, indicating these 10,800 unique medical record numbers yielded the 16,526 visits. A total of 13,454 ED cases met criteria of no billed testing or treatment other than blood glucose measurement, immunization, or COVID-19 testing (2.7% of total patients, 81% of patients with an alcohol-related diagnosis). Most patients with a final diagnosis included were billed as a level 3 facility code (76%), although the level was likely influenced by charting variability. Most were self-pay (69%) or Medicaid (20%), tailed by commercial insurance (8%) and Medicare (3%). Of these patients, 76% were male and 24% were female.

The total facility-fee billing was $22,590,000. Specific encounter collections were not available for analysis; therefore, estimates of collections were based on the hospitals’ collection rates for all visits broken down by the payors. That is, if payor x collection rate was 30%, the contribution to the overall collections was 30% of the amount payor x was billed. Using this methodology, the estimated reimbursement based on the percentage reimbursed by payor mix was about $1.7 million (7.5% collection rate). This represents an average collection of $126 per patient encounter, less than one quarter of the average visit collection at these sites. Patients’ LOS in the ED had an average of 254 minutes and median of 240 minutes. In total, this cohort of patients occupied ED beds for 56,505 hours cumulatively, or an average of 43.2 bed hours per day for alcohol intoxication-related visits. This represented 3.14% of total bed hours used across both sites during the study period.

## DISCUSSION

Alcohol intoxication is a common ED diagnosis. However, specific data on healthcare costs, charges, and uncompensated care of alcohol intoxication alone is lacking. We sought to identify and analyze a cohort of ED patients solely presenting with acute alcohol intoxication. We found this select group accounted for approximately 3% of the ED census. Patients with alcohol intoxication have been shown to be repeat users of ED services. In one study, many have been shown to present for over 100 annual visits for alcohol intoxication.^14^ Our range included up to 130 visits for a unique medical record number within the study period. Also consistent with our findings, lack of significant or complex intervention is not unusual.

Previous literature has demonstrated that the admission rate for patients with a diagnosis of alcohol intoxication is often less than 3%, far below national average hospital admission rates from the ED.^12^ Emergency medical services has likewise identified significant impact from alcohol-related calls, with over 30% of calls in one urban area as a result of alcohol consumption.^13^ Our dataset identified close to one third with multiple visits, ranging as high as 130 visits per year in one patient. This likely underestimates the actual revisits as some may have been registered under several different registration numbers due to difficulty in obtaining correct demographic information due to intoxication.

One commonality characterizing alcohol-related visits is that they generally impose a costly public health burden on EDs. One study from Australia found higher overall costs for alcohol-positive patients compared to those with negative alcohol levels, and a high weekly cost of over $144,000; while the cost included all alcohol-positive patients it was still felt to be an underestimation per the authors.^14^ Estimates of US data found ED costs related to acute alcohol consumption to be nine billion dollars per year, although the final diagnoses were more inclusive and likely included significant evaluations for other concerns or effects of alcohol.^9^ A prior cohort study from San Diego found a collection rate of 15.4% of ED visits related to alcohol use in a specific but far smaller cohort of 409 patients arrested for public intoxication.^15^ This aligns with our findings of a very limited collection rate of 7.5% in our cohort. Clinically, the cumulative 56,505 hours of ED time is substantial. These 56,505 hours occupied ED beds with the need for safety observation given the higher risk of falls among intoxicated patients, pulling staff from completing other tasks in the ED.

Not specifically addressed in this paper are the benefits and resources required to provide both alcohol reductions and cessations services as well as counseling to patients with alcohol intoxication in the ED. Strategies that are often employed, such as SBIRT (Screening, Brief Intervention and Referral to Treatment) or other screenings, were not captured in the facility charges. Given the significant cost of care and poor collection rate, alternative strategies to address and treat alcohol intoxication are warranted. As demonstrated here, 81% of those visiting the ED solely for alcohol intoxication did not require further significant medical care.

One option, sobering centers, may represent an opportunity to decrease ED use and associated costs, when used within a specific EMS protocol.^1^ A 2017 study found costs per visit of $264.18 at a sobering center compared to an average ED cost per visit of $648.72.^16^ These centers have also demonstrated increased connectivity to further care and rehabilitation, which have further decreased downstream costs as well.^1^,^16^ Emergency medical services has demonstrated the ability to appropriately identify intoxicated individuals without other illnesses and transport them to sobering centers rather than to EDs. This highlights the capability and skillset of emergency medical technicians and paramedics—along with an evidence-based protocol or checklist criteria—to differentiate those patients who can safely consider alternative destinations outside the traditional ED.^17^–^18^

Visits to the ED may represent a unique opportunity to offer preventative care and treatment for this vulnerable population. Prior initiatives have included increased offerings of detoxification and rehabilitation programs, medication treatments, and psychosocial support from interdisciplinary teams.^19^–^20^ Yet these have also been found to be successful in sobering centers.^1^ Given the high costs, inconsistent uptake, resource needs, and variable success of these programs in EDs, alternative destinations may prove superior for the right cohort.

## LIMITATIONS

It must be noted that this review included only facility fees. Due to current limitations of available data, professional fees were not reviewed. Future analysis is warranted to compare these collection rates against professional fees for this demographic. Unfortunately, individual patient-level collections were not available retrospectively due to a billing-related software change since the study period. As the encounter-level collections were not available for review, the $1.7 million in collections was estimated by the known collection rates specific to each of the respective insurances and weighted based on the respective prevalences. The collection rates may differ slightly for this specific demographic, but we believe they would likely be lower or equal to the general collection rates. Lastly, this cohort was identified by excluding encounters with mixed intoxications or co-ingestions. Further analysis of this stratification and/or review of a similar demographic presenting for drug use may be warranted given the similar patterns of resource utilization.

## CONCLUSION

Patients presenting with acute alcohol intoxication and no other complaints comprise a relatively small minority of ED visits, yet they result in substantial charges and ED resources. Of all ED visits, 2.7% were attributed to this cohort, and they occupied 3.14% of all ED bed hours. Based on the known facility collection rates per insurer, the weighted prevalence of insurers among this cohort yields an estimated overall facility collection rate of 7.5%. In general, self-pay, the source of most patients, is collected at a rate and amount markedly below all other payors. Opportunities to provide proven alcohol-related interventions should consider the unreimbursed costs of ED patients with intoxication when considering cost effectiveness.

## Figures and Tables

**Figure 1:**
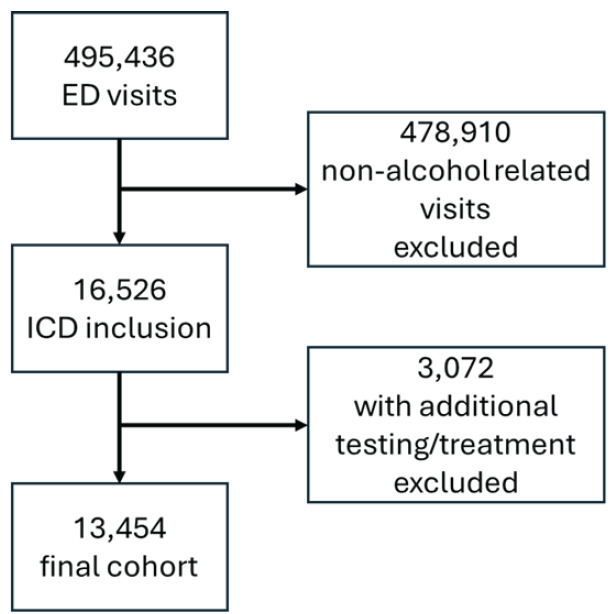
Flow diagram for cases included. *ED*, emergency department; *ICD*, *International Classification of Diseases.*

**Table 1:** *International Classification of Diseases* codes and respective descriptors used for inclusion.

ICD 10 Code	Primary Dx Description
F10.10	Alcohol abuse, uncomplicated
F10.120	Alcohol abuse with intoxication, uncomplicated
F10129	Alcohol abuse with intoxication, unspecified
F10.20	Alcohol dependence, uncomplicated
F10.220	Alcohol dependence with intoxication, uncomplicated
F10.229	Alcohol dependence with intoxication, unspecified
F10.920	Alcohol use, unspecified with intoxication, uncomplicated
F10.929	Alcohol use, unspecified with intoxication, unspecified

*ICD-10*, *International Classification of Diseases, 10^th^ Rev; dx, diagnosis.*
